# Growth and development of skeletal anomalies in diploid and triploid Atlantic salmon (*Salmo salar*) fed phosphorus-rich diets with fish meal and hydrolyzed fish protein

**DOI:** 10.1371/journal.pone.0194340

**Published:** 2018-03-22

**Authors:** Stefano Peruzzi, Velmurugu Puvanendran, Guido Riesen, Rudi Ripman Seim, Ørjan Hagen, Silvia Martínez-Llorens, Inger-Britt Falk-Petersen, Jorge M. O. Fernandes, Malcolm Jobling

**Affiliations:** 1 Faculty of Biosciences, Fisheries and Economics, University of Tromsø -the Arctic University of Norway, Tromsø, Norway; 2 Department of Production Biology, Nofima AS, Tromsø, Norway; 3 Skretting Aquaculture Research Center, Stavanger, Norway; 4 SalmoBreed AS, Bergen, Norway; 5 Faculty of Biosciences and Aquaculture, Nord University, Bodø, Norway; 6 Aquaculture and Biodiversity Research Group, Institute of Science and Animal Technology (ICTA), Universitat Politècnica de València, València, Spain; Universidade de Vigo, SPAIN

## Abstract

Diploid and triploid Atlantic salmon, *Salmo salar* were fed high-protein, phosphorus-rich diets (56–60% protein; ca 18g phosphorus kg^-1^ diet) whilst being reared at low temperature from start-feeding until parr-smolt transformation. Performances of salmon fed diets based on fish meal (STD) or a mix of fishmeal and hydrolysed fish proteins (HFM) as the major protein sources were compared in terms of mortality, diet digestibility, growth and skeletal deformities. Separate groups of diploids and triploids were reared in triplicate tanks (initially 3000 fish per tank; tank biomass ca. 620 g) from 0–2745 degree-days post-start feeding (ddPSF). Growth metrics (weight, length, condition factor) were recorded at ca. 4 week intervals, external signs of deformities to the operculum, jaws and spinal column were examined in parr sampled at 1390 ddPSF, and external signs of deformity and vertebral anomalies (by radiography) were examined in fish sampled at the end of the trial (2745 ddPSF). The triploid salmon generally had a lower mass per unit length, i.e. lower condition factor, throughout the trial, but this did not seem to reflect any consistent dietary or ploidy effects on either dietary digestibility or the growth of the fish. By the end of the trial fish in all treatment groups had achieved a weight of 50+ g, and had completed the parr-smolt transformation. The triploids had slightly, but significantly, fewer vertebrae (Triploids STD 58.74 ± 0.10; HFM 58.68 ± 0.05) than the diploids (Diploids STD 58.97 ± 0.14; HFM 58.89 ± 0.01), and the incidence of skeletal (vertebral) abnormalities was higher in triploids (Triploids STD 31 ± 0.90%; HFM 15 ± 1.44%) than in diploids (Diploids STD 4 ± 0.80%; HFM 4 ± 0.83%). The HFM diet gave a significant reduction in the numbers of triploid salmon with vertebral anomalies in comparison with the triploids fed the STD diet possibly as a result of differences in phosphorus bioavailability between the two diets. Overall, the incidence of skeletal deformities was lower than reported in previous studies (Diploids 20+%, Triploids 40+%), possibly as a result of the combination of rearing at low-temperature and phosphorus-rich diets being used in the present study.

## Introduction

The induction of triploidy represents the most common and reliable method for the production of sterile fish. Efforts to produce functionally sterile stocks relate to the potential association with improved post-pubertal somatic growth, survival and flesh quality [1]. Sterility is also a management tool for the genetic containment of cultured fish in the event of accidental escapes or targeted stocking and introductions into natural waters [1, 2].

Triploid Atlantic salmon (*Salmo salar*) are often reported to have a higher incidence of skeletal deformities than diploids [3–6], and there is evidence that the proportions of triploid fish with skeletal abnormalities can be reduced by changing diet formulations [7–8]. In particular, triploids may have higher dietary requirements for phosphorus and protein than diploids, and there may also be differences in amino acid requirements and metabolism [7–11]. There are also dissimilarities in the morphology of the digestive system of diploid and triploid Atlantic salmon [12] and these might have an influence on digestion and absorption, and subsequent nutrient utilization and growth.

Hydrolyzed fish proteins have high concentrations of free amino acids and low molecular peptides that may be absorbed relatively easily, and these could have a subsequent influence on nutrient utilization and fish growth [13]. Protein hydrolysates produced from fish and fish by-products have been used as feed ingredients for a number of farmed species [13], including Atlantic salmon [14].

In this study, the performances of diploid and triploid salmon reared at low temperature and fed a high-protein phosphorus-rich fishmeal-based diet were compared with those of salmon fed a similar diet containing a high proportion of fish protein hydrolysate. Fish were examined at regular intervals, from start-feeding until the completion of parr-smolt transformation, with records being made of survival, growth, and skeletal deformities. Expectations were that the combination of low-temperature rearing and feeding a high-protein phosphorus-rich diet containing hydrolysed fish proteins might alleviate some of the problems associated with the development of skeletal deformities in triploid salmon.

## Materials and methods

### Fish and rearing conditions

On 20 August 2015, eggs from twenty female Atlantic salmon were fertilized with milt from thirteen males (Stonfiskur, SalmoBreed AS, Iceland), each female being crossed with either one or two males resulting in full-sib and half-sib families. At 300° min post-fertilization at 5°C, half of the fertilized eggs from each female was subjected to a hydrostatic pressure shock (TRC-APV; Aqua Pressure Vessel, TRC Hydraulics Inc., Dieppe, NB, Canada) of 9500 psi applied for 5 min [15] to induce triploidy. The remaining eggs served as diploid controls, giving 40 groups in total (20 diploid and 20 triploid). On 04 November 2015 the eyed-eggs (ca. 400 degree-day, dd) were shipped to the Tromsø Aquaculture Research Station, Norway (69°N, 19°E). Diploid and triploid families were held in separate incubation trays (n = 40) in a flow-through system at an average temperature of 4.8°C (minimum: 3.9°C, maximum: 5.8°C) following standard rearing procedures. Hatching was completed on 02 December 2015. Ploidy status of the fish was verified by flow cytometry [16] using 20 and 50 newly-hatched fry from each diploid and triploid family, respectively. Three out of the twenty triploid families had small percentages (2–5%) of diploid fry and were discarded along with their diploid counterparts.

Just prior to start-feeding (18 February 2016), equal numbers of individuals for each ploidy within each of the 17 families were pooled and allocated to twelve 200 L circular indoor tanks (ca. 3000 fish per tank, and tank biomass ca. 620 g). Triplicate tanks per ploidy were fed a standard fish meal (STD) diet or a modified diet in which 45% of the fish meal fraction was replaced with hydrolysed fish proteins (HFM) (Table 1; Skretting AS, Stavanger, Norway). Phosphorus concentrations in STD and HFM diets were analysed (MasterLab, Boxmeer, Netherlands) to be 19 g kg^-1^ and 18 g kg^-1^, respectively, which is considerably in excess of the reported requirement of Atlantic salmon (8 g phosphorus kg^-1^ diet) [17]. An inert marker (yttrium) was added to the largest pellet (3 mm) of each diet (10 g kg^-1^) for analysis of dietary digestibility.

**Table 1:** Formulation and chemical composition of the diets.

Pellet size (mm)	STD diet			HFM diet		
	0.5–1.0	1.2	1.5–3.0	0.5–1.0	1.2	1.5–3.0
Wheat	7.2	6.1	6.9	5.4	5.5	6.9
Wheat gluten	10	10	10	10	10	10
Soy Protein Concentrate (SPC)	14.4	16.7	17.9	14	16.2	16.7
NA Fishmeal	55	55	50	30	30	27.5
CPSP Special G—hydrolyzed FM	0	0	0	25	25	22.5
Fishoil Nordic	11	10.8	11.6	9.4	9.2	10.2
Water	0	0	0.4	1.6	0.9	1.5
Yttrium premix*	0	0	0.1	0	0	0.1
Premix (Minerals, Vitamins, Amino acids)	2.4	1.4	3	4.7	3.3	4.6
TOTAL	100	100	100	100	100	100
Chemical composition (%)						
Moisture	7.9	7.5	7.2	7.9	7.5	7.1
Protein	55.9	56.8	56.0	60.3	59.0	56.9
Fat	17.7	18.3	19.1	17.3	18.8	19.6

Fish meal (STD) and hydrolyzed fish protein (HFM) diets (Skretting AS, Stavanger, Norway).

*Marker Yttrium used in 3.0mm diets only. CPSP = Fish meal hydrolysate Special-G (SoproPêche. Boulogne-sur-Mer. France)

**Fig 1:**
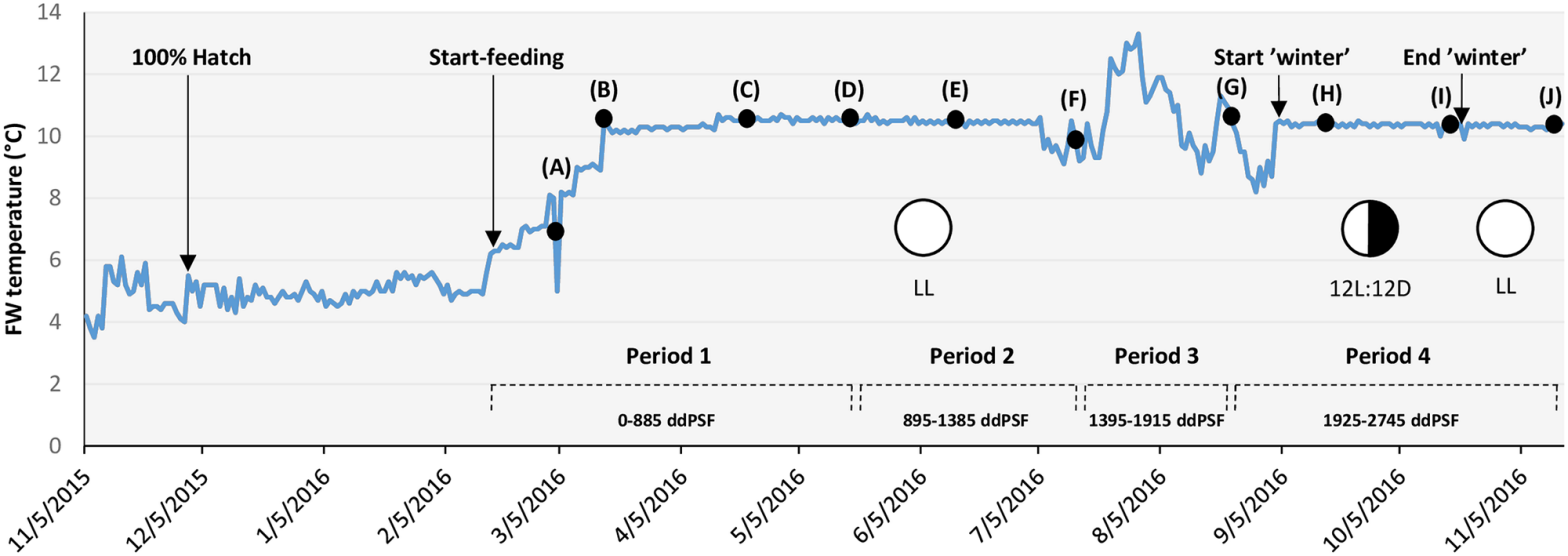
Overview of rearing conditions and sampling points during the trial. Periods 1–4 cover 0–2745 degree-days post start-feeding (ddPSF). Fish growth expressed as the Thermal Growth Coefficient (TGC) was calculated from bulk weight increase during each period. Black dots indicate intermediate sampling points where 25 fish per tank were weighed and measured. Arrows indicate other events or operations. FW = freshwater; LL = constant light; 12L:12D = light regime used during ‘winter’ stimulation. Sampling points: (A) 95 ddPSF; (B) 218 ddPSF; (C) 580 ddPSF; (D) 875 ddPSF; (E) 1168 ddPSF; (F) 1455 ddPSF; (G) 1888 ddPSF; (H) 2090 ddPSF; (I) 2454 ddPSF; (J) 2745 ddPSF.

Feed was delivered via electrically driven disc feeders programmed to supply 6–9 meals each day, and the amount of feed provided was always in excess of that consumed. At the onset of start-feeding, water temperature was gradually increased to 10°C over a period of 4 weeks and was maintained at this level using heated water (10.0 ± 0.5°C), except during the summer (04 July– 02 September) when fish were exposed to ambient water temperature (range 9.5–12.5°C) (Fig 1). General husbandry conditions followed standard in house procedures for Atlantic salmon. Fish were transferred from 200 L to 500 L tanks on 20 May 2016. Fish were reared under constant light (LL) throughout the experiment except for the period 06 September—18 October when 12h light (12L:12D) was used to simulate winter conditions and induce parr-smolt transformation (Fig 1).

Dissolved oxygen was measured regularly, and levels in the outlet water never fell below 80% saturation. Dead fish were removed each day, counted and mortality recorded. Fish had completed parr-smolt transformation by the end of the experiment on 15 November 2016.

### Fish growth

Fish growth in each tank was calculated from biomass increase during the course of four periods (Period 1–4) covering 0–2745 degree-days post start-feeding (ddPSF) (Fig 1). At these times biomass re-adjustments were made to maintain tank biomass below 40–45 kg m^-3^. The total weight of fish (biomass) in each tank was recorded and the mean body weight (MW g) and numbers of fish present estimated by taking three random subsamples of 50 fish from each tank. Fish growth was assessed using the thermal growth coefficient (TGC) following Cho [18]:

$$TGC=1000\frac{{{MW}_{1}}^{\frac{1}{3}}-{{MW}_{0}}^{\frac{1}{3}}}{\sum degree-days}$$

where MW_0_ and MW_1_ represent mean fish weights at the start and end of a growth period.

An estimate of growth was also obtained by measuring individual body weight (IW g) and fork length (FL cm) of 25 fish per tank at ca. 4 week intervals, beginning at 2 weeks after start-feeding (95 ddPSF) (Fig 1). Sampled fish were euthanized with an overdose (120 mg L^-1^) of anaesthetic (Benzocaine) prior to measurement. Fish condition factor (K) was calculated as:

$$K=100\;({WL}^{-3})$$

where W is body weight (g) and L is the fork length (cm).

### Visual inspection and radiography

**Fig 2:**
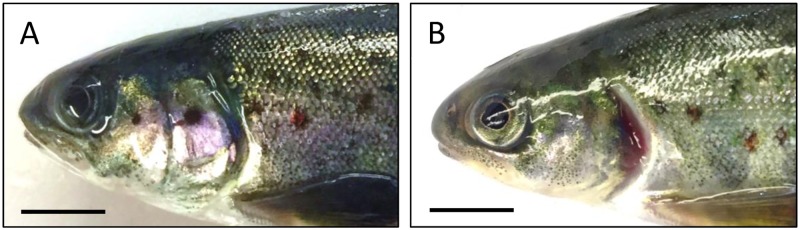
Lateral view of the head region of fish at parr stage. View of anterior (cranial-trunk) of fish at parr stage (1390 ddPSF). (A) normal operculum and (B) moderate opercular shortening. Scale bars represent 1cm.

At the parr stage (1390 ddPSF), 100 fish per tank were visually inspected for externally detectable deformities to the operculum (Fig 2), jaws (i.e. misalignment, shortening, downward curving) and spinal column. At the end of the experiment (2745 ddPSF), 40 fish from each tank (n = 120 fish/group) were inspected for externally detectable deformities and then radiographed to detect lower jaw deformity and skeletal (vertebral) anomalies (i.e. number, type and location). For visual inspections and radiological examinations, fish were euthanized with an overdose of anaesthetic (Benzocaine, 120 mg L^-1^) and then frozen (-20°C). Frozen fish were allowed to thaw and were fully stretched before observation for detection of deformed opercula or being X-rayed. For this purpose, 10 fish were placed on a 35x43 cm digital plate with a GP flexible phosphorus screen (Direct View CR500, Carestream Health Inc., USA) and exposed twice for 4 sec at 3 mA and 60 kV using a Nanodor 2 X-ray apparatus (Siemens, Germany). The plates were digitally scanned (Direct View CR500, Carestream Health Inc., USA) and pictures examined using image analysis (ImageJ 1.49v, Wayne Rasband, National Institutes of Health, USA). The spinal column was divided into four regions (R) according to the classification of Kacem et al. [19]: R1 (cranial trunk) covering vertebrae (V) V1-V8, R2 (caudal trunk) covering vertebrae V9-V30, R3 (tail) covering V31-V49, and R4 (tail fin) covering V50-V58 to V60. Individuals with at least one deformed vertebra were classified as deformed and the type of deformity was identified following Witten et al. [20]. Numbers of vertebrae were recorded for each fish and when two or more fused vertebrae were seen each was counted. Deformities of the spinal column were further characterized by measuring the ratio between the distance (pixels) from the tip of the head to the posterior edge of the dorsal fin, and from the posterior edge of the dorsal fin to the caudal peduncle as index of shortened trunk (STR) and shortened tail area (STA) following Taylor et al. [21].

Lower jaw deformations were recorded and assessed by calculating the lower jaw index (LJI) [6,22] as:

$$LJI=\frac{L2}{L1}$$

where L1 and L2 represent the distance (pixels) between the articulation point of the pectoral fin and the tips of the upper and lower jaws, respectively.

### Dietary digestibility

Apparent digestibility coefficients (ADCs) of dietary protein, fat, phosphorus and calcium were measured at the end of the experiment. For this purpose, 5 fish per tank were sacrificed, gastrointestinal tracts were removed following dissection and digesta from the rectum transferred into Eppendorf tubes, and frozen at −80°C until analysis. Diets and faecal samples were analysed for dry matter, crude fat, crude protein, phosphorus, calcium and Yttrium.

Chemical compositions of diets and faeces were analysed for dry matter (DM, 105°C overnight), crude protein (Kjeldahl-N×6.25), crude fat (HCl hydrolysis followed by petroleum ether extraction), and ash (550°C 24h). Yttrium was determined in diets and faeces using an atomic absorption spectrometer (Perkin Elmer 3300, Perkin Elmer, Boston, MA, USA) after nitric acid/hydrochloric acid digestion. Phosphorus and calcium were analysed in diets and faeces by atomic emission spectrometry (ICP-OES) after hydrochloric and nitric acid digestion. The apparent digestibility coefficients for protein, fat, phosphorus and calcium were calculated using the following formula:

$$ADC[\%]=100[1-(\frac{F}{D}\;\frac{DY}{FY})]$$

Where F is the percentage of nutrient in faeces, D is the percentage of nutrient in the diet, DY is the percentage of Yttrium in the diet and FY is the percentage of Yttrium in faeces [17].

### Ethical statement

This study was carried out in accordance with the Norwegian regulations for use of animals in experiments and was approved by the Norwegian Committee on Ethics in Animal Experimentation via project licence (Permit ID 8180) issued by the Norwegian Food Safety Authority (Mattilsynet, FOTS). The growth trial was carried out in an approved facility (Tromsø Aquaculture Research Station, FOTS licence nr. 110) by trained and licensed personnel. Terminal measurements were performed on fish euthanized with an overdose of anaesthetic (Benzocaine, 120 mg L^-1^). All efforts were made to minimize fish suffering.

### Statistical analysis

Data expressed as percentages were arcsine-transformed prior to analysis, and data were checked for normality (Shapiro-Wilk test), independence and homogeneity of variances (Levene’s test) to satisfy the assumptions of ANOVA. Data for mortality, thermal growth coefficient (TGC), opercular shortening, jaw and skeletal anomalies from radiological examinations, and dietary digestibility (ADC) were analysed using two-way ANOVA with ploidy and diet as fixed factors. Ploidy and diet effects on length, weight and condition factor were tested using a nested ANOVA, with tank as random factor nested in ploidy and diet groups. When significant differences were found, Tukey’s post-hoc multiple-comparison test using least square means was used to examine for differences between individual treatments. Group-wise differences in the number of vertebrae were tested by non-parametric Kruskal–Wallis test. All analyses were performed using SYSTAT 13 version 13.1 (SYSTAT Software Inc.) and results were considered significant at *P*≤0.05. Results are reported as mean ± standard error of the mean (SEM).

## Results

### Fish mortality

**Fig 3:**
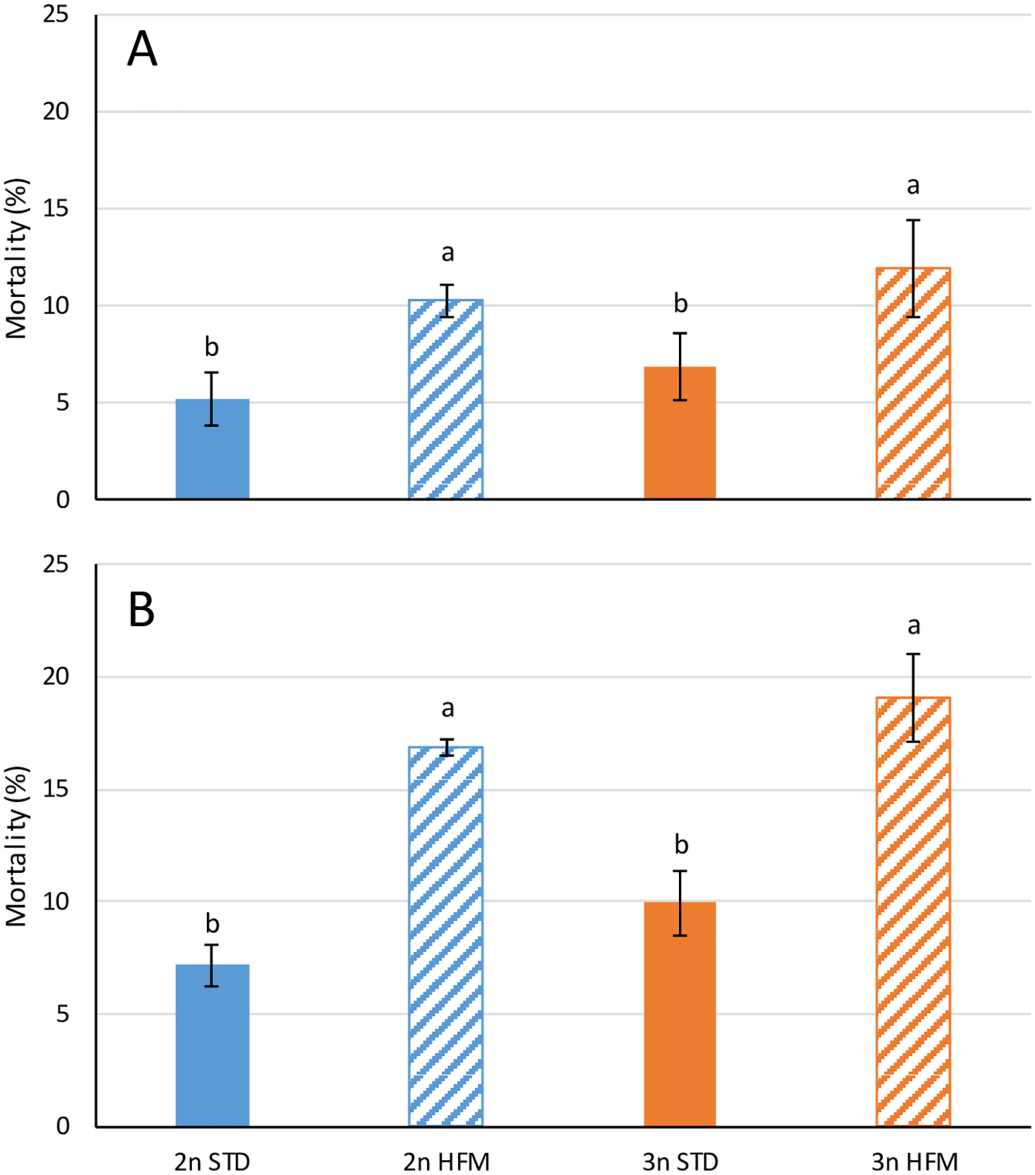
Fish mortality. Percentage of dead fish recorded in diploid (2n) and triploid (3n) salmon fed fish meal (STD) and hydrolyzed fish protein (HFM) diets over (A) the initial period (0–885 ddPSF) and (B) the whole experiment (0–2745 ddPSF). Different letters denote significant differences (P<0.05). Data are presented as mean ± SEM (n = 3).

During the initial rearing period (Period 1, Fig 1; 0–885 ddPSF), mortality was higher (*P*<0.001) in fish fed the hydrolyzed fish protein (HFM) diet than in those fed the standard diet (STD) (Fig 3A), but there were no differences between ploidy groups within diets. The same trend was observed during the entire experiment (0–2745 ddPSF), with cumulative mortality being higher (*P*<0.001) in fish fed the HFM diet than in those fed the STD diet, but with no significant differences between diploid and triploid fish (Fig 3B).

### Fish growth

**Fig 4:**
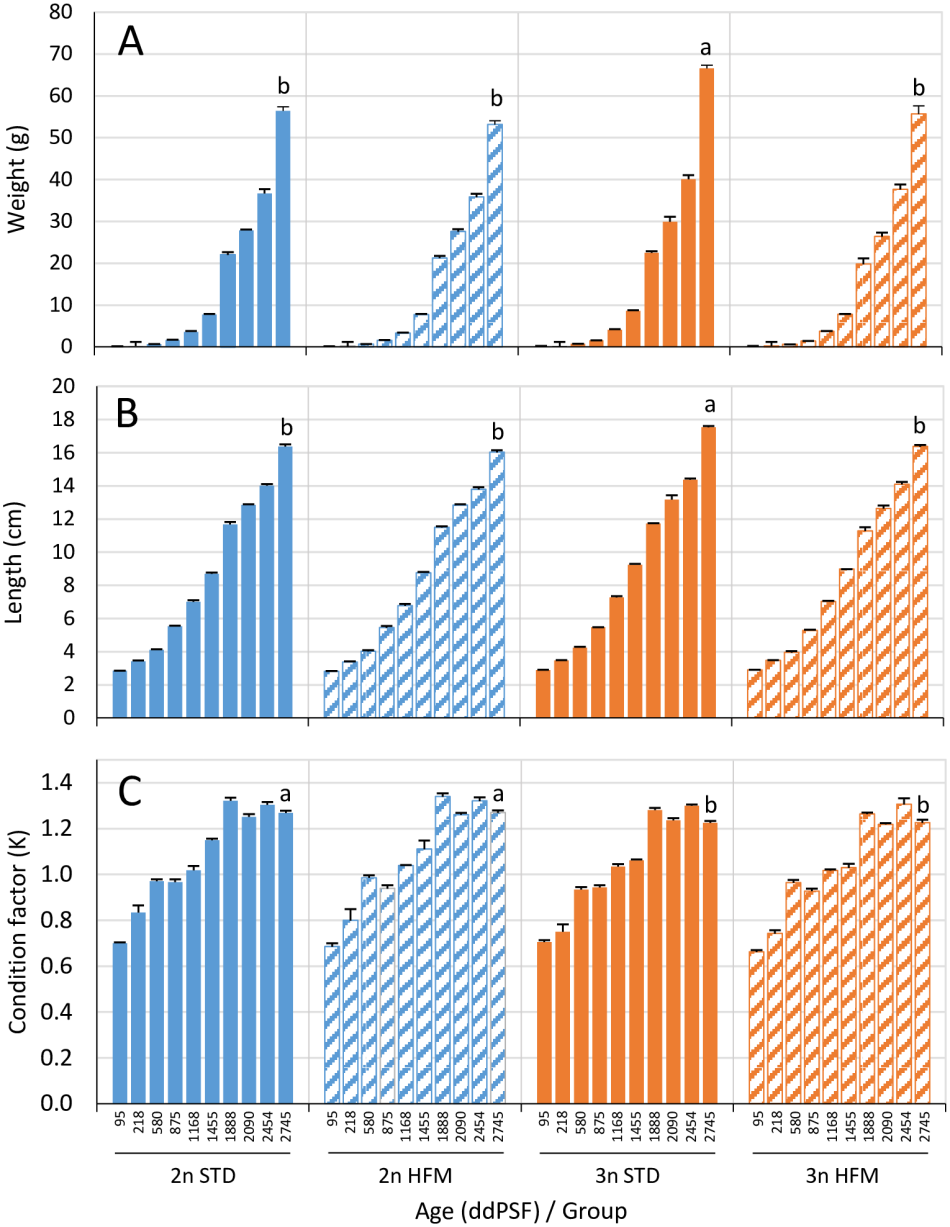
Fish growth from individual measurements. (A) Body weight, (B) length and (C) condition factor of diploid (2n) and triploid (3n) Atlantic salmon, *Salmo salar*, fed fish meal (STD) and hydrolyzed fish protein (HFM) diets measured at ten sampling points (see Fig 1: 95–2745 ddPSF). Results from statistical comparisons among groups are shown for the last sampling point (2745 ddPSF) (for full overview see Supplementary material: S2 Table). Different letters denote significant differences (*P*<0.05). Data are presented as means ± SEM (n = 3).

There were no clear and consistent effects of either ploidy or diet on the growth trajectories of the fish (Table 2 and Fig 4)(S2 Table). During the final study period (1925–2725 ddPSF), there was a significant effect (two-way ANOVA) of both ploidy (*P* = 0.029) and diet (*P* = 0.010) on the growth of the fish with triploids fed the STD diet performing better than all other groups (Table 2). By the end of the trial (2745 ddPSF) the triploids given the STD diet were both heavier (Fig 4A) and longer (Fig 4B) than fish in the other treatments (*P*<0.001). At this time the diploid fish had higher (*P*<0.01) condition factors (K) than the triploids (Fig 4C).

**Table 2:** Fish growth assessed using thermal growth coefficient.

Period (ddPSF)	Group				*P*-values*		
	2n STD	2n HFM	3n STD	3n HFM	Ploidy	Diet	Interaction
0–885	0.84±0.01	0.80±0.03	0.79±0.01	0.78±0.01	0.073	0.133	0.367
895–1385	1.14±0.05^b^	1.13±0.06^b^	1.25±0.01^a^	1.31±0.03^a^	***<0*.*01***	0.543	0.407
1395–1915	1.78±0.09	1.92±0.05	1.70±0.12	1.77±0.09	0.263	0.290	0.724
1925–2745	1.29±0.04^b^	1.17±0.01^b^	1.59±0.10^a^	1.25±0.09^b^	***0*.*029***	***0*.*010***	0.150

Thermal growth coefficient (TGC) of diploid (2n) and triploid (3n) Atlantic salmon, *Salmo salar*, fed fish meal (STD) and hydrolyzed fish protein (HFM) diets calculated for four periods covering 0–2745 ddPSF (see Fig 1). Data are presented as means ± SEM (n = 3).

### Fish deformities—External examination

**Fig 5:**
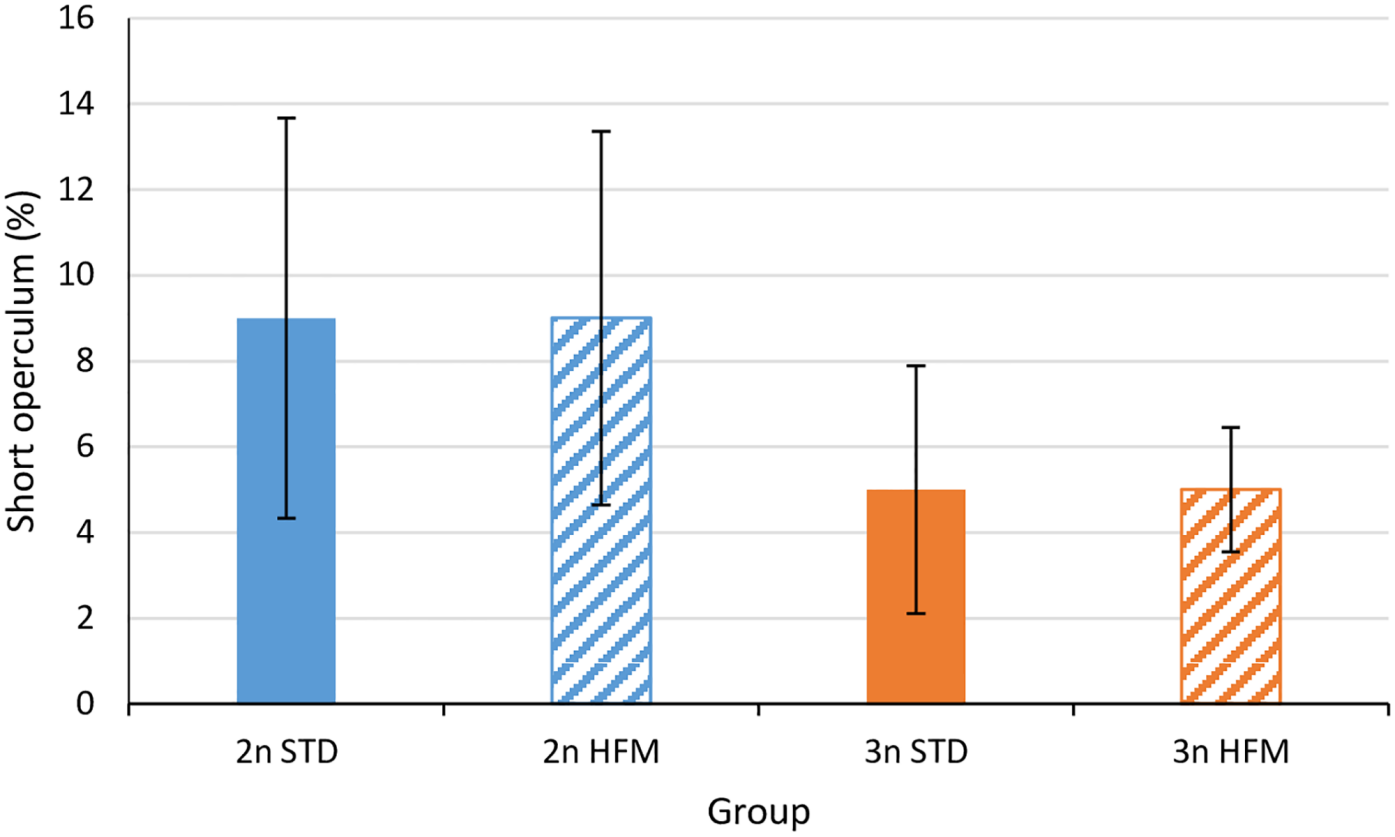
Percentage of short opercula in salmon parr. Percentages of short opercula in diploid (2n) and triploid (3n) Atlantic salmon, *Salmo salar*, parr (1390 ddPSF) fed fish meal (STD) and hydrolyzed fish protein (HFM) diets. Data are presented as mean ± SEM (n = 3).

At the parr stage (1390 ddPSF) shortening of the operculum (Fig 2) was observed in fish of both ploidies, but no significant treatment effects were seen (Fig 5). At the end of the trial (2745 ddPSF), none of the sampled fish had short opercula (S1 Table) suggesting that recovery may have occurred. At both times, externally detectable jaw or spinal deformities were low (≤2%) irrespective of ploidy and diet and the data were not subjected to detailed statistical analysis (S1 Table).

### Fish deformities—Radiological examination

**Fig 6:**
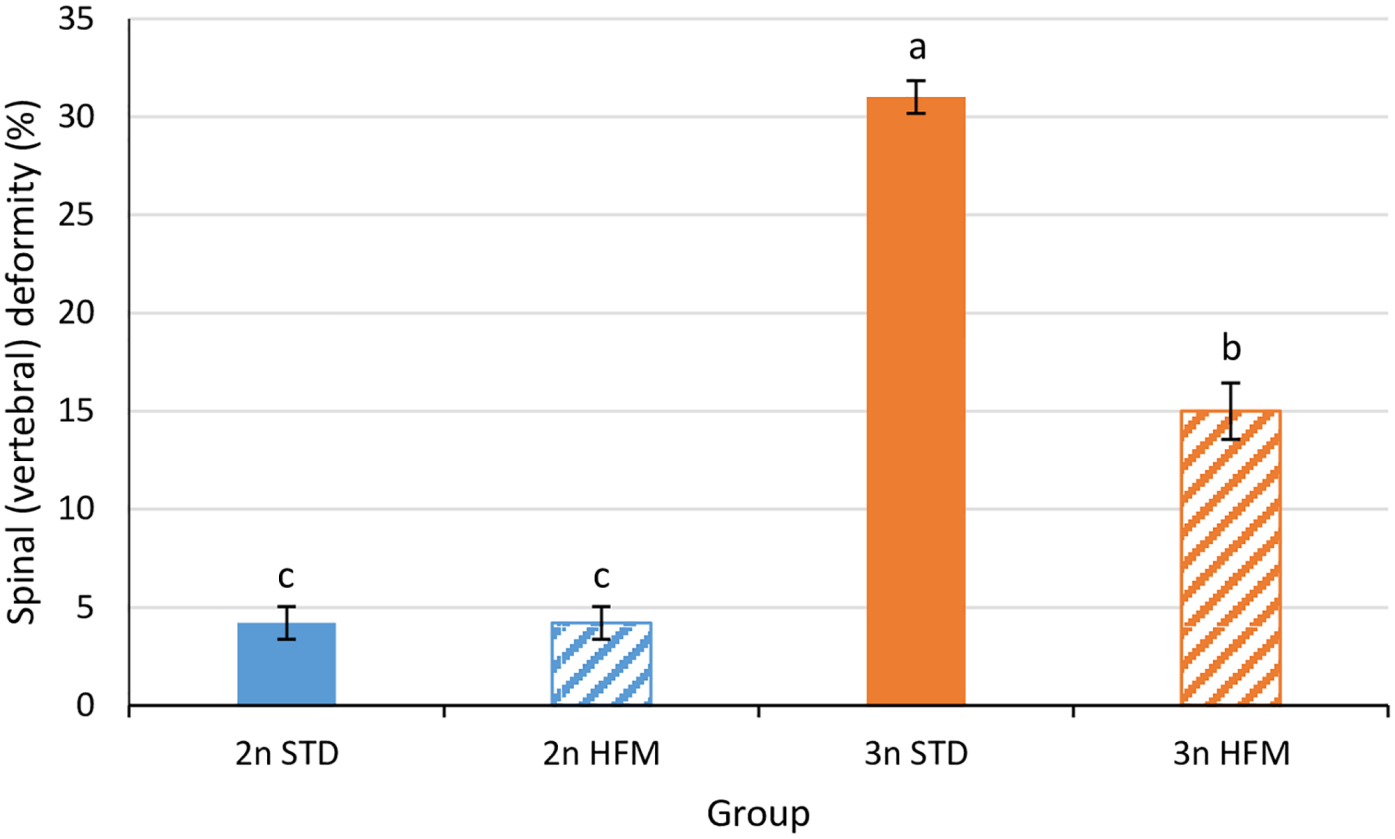
Percentage of vertebral deformities in salmon smolt detected by radiography. Percentages of fish with spinal (vertebral) deformity among diploid (2n) and triploid (3n) Atlantic salmon, *Salmo salar*, smolt (2745 ddPSF) fed fish meal (STD) and hydrolyzed fish protein (HFM) diets. Different letters denote significant differences (*P*<0.05). Data are presented as mean ± SEM (n = 3).

**Fig 7:**
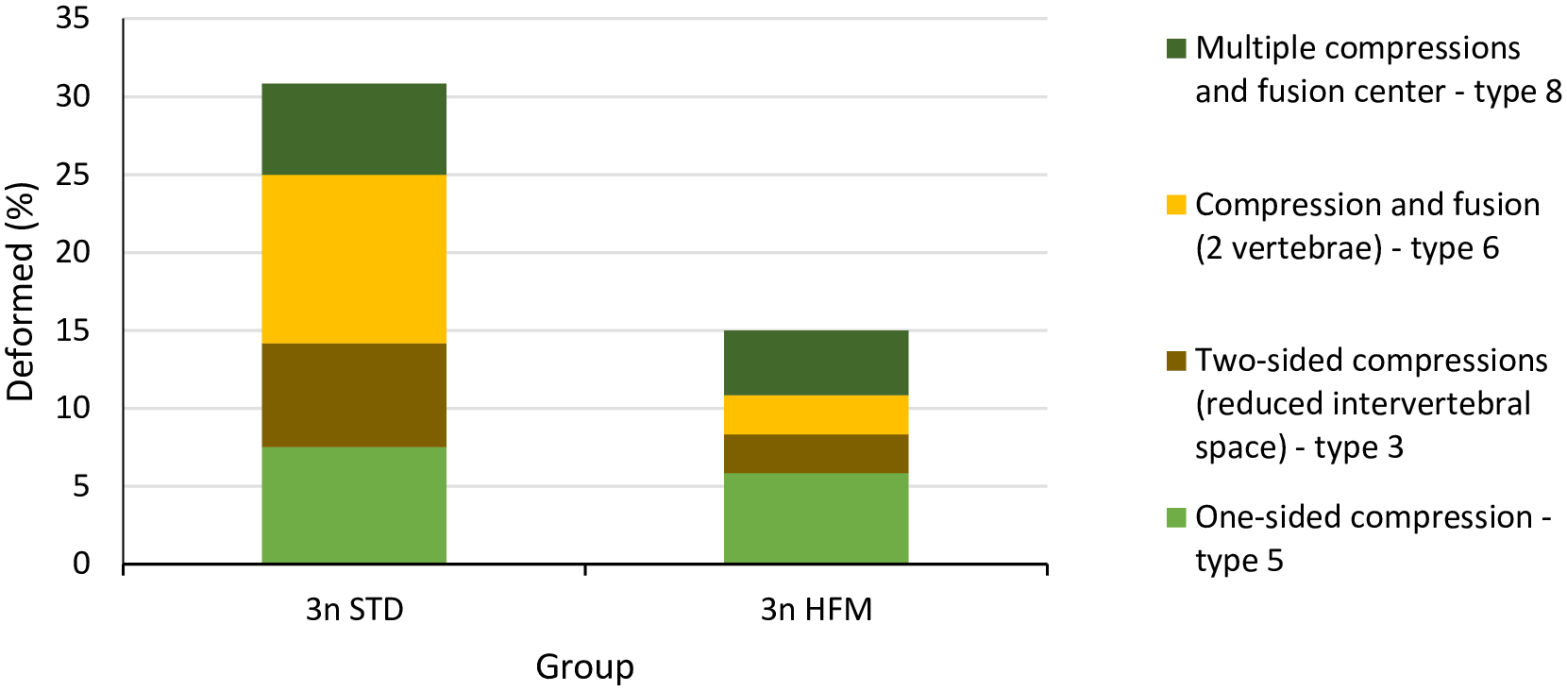
Types of vertebral deformities observed in triploid salmon smolt. Mean prevalence of vertebral deformity types in triploid (3n) Atlantic salmon, *Salmo salar*, smolt (2745 ddPSF) fed fish meal (STD) and hydrolyzed fish protein (HFM) diets. Classification of vertebral deformities according to Witten et al. [20].

**Fig 8:**
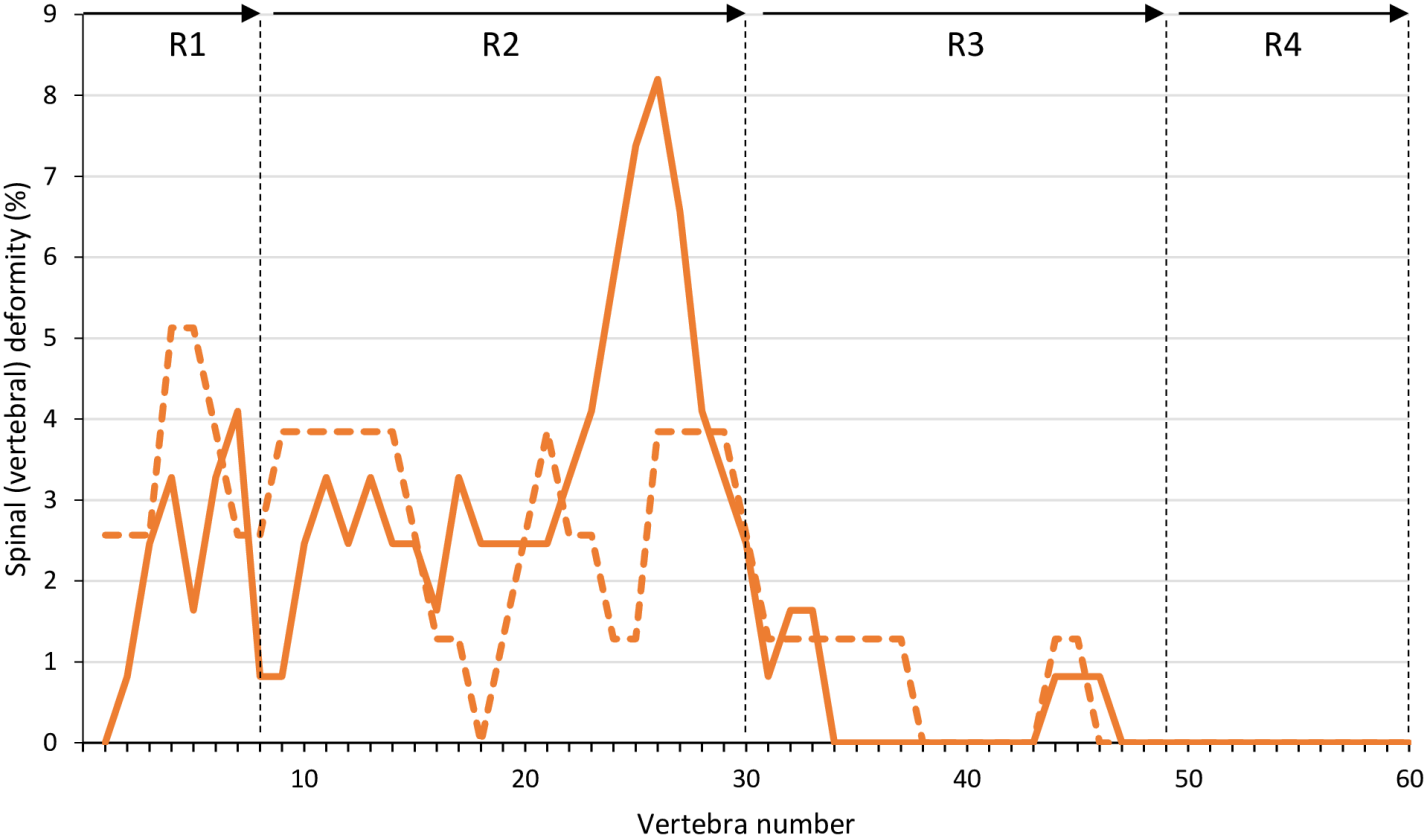
Occurrence of deformed vertebrae in triploid salmon smolt. Percentage of deformed vertebrae in different regions (R1-R4) of the spinal column of triploid Atlantic salmon, *Salmo salar*, smolt (2745 ddPSF) fed fish meal (STD) (solid line) and hydrolyzed fish protein (HFM) (dashed line) diets. Vertebral regions according to Kacem et al. [19]; R1 = cranial trunk, R2 = caudal trunk, R3 = tail region and R4 = tail fin.

There were significant diet and ploidy effects on vertebral numbers, skeletal deformities and abnormalities (Fig 6 and S1 Table). There were slightly, but significantly, fewer vertebrae in triploids (STD diet, 58.74 ± 0.10; HFM diet, 58.68 ± 0.05) than in diploids (STD diet, 58.97 ± 0.14; HFM diet, 58.89 ± 0.01). Triploid fish had a significantly higher (*P*<0.001) incidence of spinal (vertebral) deformities than the diploids (Fig 6), and vertebral anomalies of four types were recorded (Fig 7). These were type 3 (two-sided compression and reduced intervertebral space), type 5 (one-sided compression), type 6 (compression and fusion), and type 8 (multiple fusion center) [20]. Overall, vertebral compressions with or without fusion were equally represented within each diet group. Detailed examination of the triploid fish revealed that the preponderance of deformed vertebrae occurred in the more anterior regions of the spinal column (Regions 1–2; vertebrae 1–30) with vertebra V26 being the most affected in fish fed the STD diet (Fig 8). The incidence of vertebral deformities was lower (*P*<0.001) in the triploids given the HFM diet than in those fed the STD diet, but was still significantly higher (*P*<0.001) than in diploids fed either the STD or the HFM diet (Fig 6).

### Dietary digestibility

Protein ADC was higher (*P*<0.001) in diploid than in triploid fish for both diets (Table 3). Digestibility of dietary lipid was unaffected by ploidy or diet but a significant (*P*<0.001) interaction was observed (Table 3). Diploid fish fed the hydrolyzed fish protein (HFM) diet had higher fat ADC than those fed the fish meal (STD) diet, whereas the opposite was observed for triploids. Phosphorus ADCs were high (range: 85–89%) with a significant (*P*<0.01) effect of diet but not ploidy. Phosphorus digestibility was lowest (85%) in triploids fed the STD diet. There were no significant effects of either ploidy, diet or their interaction on calcium digestibilities.

**Table 3:** Apparent digestibility of diets.

Nutrient ADC (%)	Group				*P*-values*		
	2n STD	2n HFM	3n STD	3n HFM	Ploidy	Diet	Interaction
Protein	90.32±0.14^a^	90.45±0.41^a^	88.20±0.53^b^	88.52±0.15^b^	***<0*.*001***	0.543	0.798
Fat	95.21±0.11^b^	96.61±0.16^a^	96.79±0.09^a^	95.08±0.20^b^	0.886	0.311	***<0*.*001***
P	87.31±0.64^a,b^	89.17±1.29^a^	84.67±0.52^b^	88.95±0.10^a^	0.099	***<0*.*01***	0.151
Ca	77.11±2.86	78.66±2.88	80.27±1.39	77.72±1.16	0.632	0.829	0.382

Apparent digestibility coefficients (ADCs) for protein, fat, phosphorus (P) and calcium (Ca) in diploid (2n) and triploid (3n) Atlantic salmon, *Salmo salar*, fed fish meal (STD) and hydrolyzed fish protein (HFM) diets. Data are presented as means ± SEM (n = 3).

Different superscript letters denote significant differences.

*Values in bold and italics indicate a significant effect (two-way ANOVA, *P* < 0.05) of diet, ploidy or interaction between diet and ploidy.

## Discussion

In the present study, mortality did not differ between the diploid and triploid salmon in the period from first-feeding to completion of the parr-smolt transformation. In several previous studies mortality has been reported to be higher for triploid than diploid salmon during the early life-history stages, but the differences are largely associated with the egg incubation period [2,5,23,24]. From start-feeding until completion of the parr-smolt transformation, survivorship of both diploids and triploids is generally high, and mortality is usually below 10% with no significant differences between ploidies [2,5,23,25]. As such, the mortality observations for the fish given the STD diet in the present study resemble those reported in several previous investigations on diploid and triploid salmon, but the mortality registered for the fish given the HFM diet seems to be higher than the norm. The reason for this is not known but we could speculate that the high early-stage mortality in the fish given the HFM diet was related to poorer water stability and binding in the HFM diet than the STD diet. This could have reduced the availability of feed to the fish given the HFM diet, leading to chronic underfeeding and malnutrition even though the fish appeared to be fed in excess.

The triploid salmon had slightly, but significantly, fewer vertebrae than the diploids. In addition, the prevalence of skeletal deformities was higher in the triploid salmon than in the diploids, irrespective of the dietary treatment. As such, these findings are in agreement with those of a number of previous studies on diploid and triploid Atlantic salmon reared in fresh water [2–7]. There are, however, a number of differences between observations made in the present study and those reported previously. For example, both Fraser et al. [4] and Amoroso et al. [5] reported that the highest prevalence of anomalous vertebrae in triploid salmon occurred in the caudal region (V50-V60), whereas the incidence of vertebral abnormalities was highest in the anterior regions of the spinal column in the present study. It has been suggested that a high incidence of vertebral deformities in the caudal region may be linked to dietary phosphorus deficiency [5]; if this is the case it could provide an explanation for the different findings across studies [4,5,7], including the present work. In our study all diets contained high concentrations of phosphorus, and bioavailability, i.e. high ADC, (Table 3) appeared to be high. Similarly, Fjelldal et al. [7] reported a relatively high incidence of caudal vertebral deformities in salmon fed a low-phosphorus (7.1 g phosphorus kg^-1^ diet) but not in fish fed a high-phosphorus (16.3 g phosphorus kg^-1^ diet) diet. In the same work, triploid smolt fed a high-phosphorus diet mostly suffered from deformity types (e.g. type 5 and 8) occurring in the most cranial regions (V1-V30) of the vertebral column. This is in general agreement with the findings of the present study.

In several previous studies the proportions of salmon with skeletal, or vertebral, abnormalities have been high; exceeding 20% for diploids and being over 40% in triploids [3,8]. In the present study, the percentages of fish with vertebral anomalies were much lower than this. In part, these differences probably relate to the temperatures used during egg incubation, start-feeding and early growth in fresh water; lower temperatures were used in the present study than in several others. High water temperatures during egg incubation, start-feeding and early growth of salmon usually results in increased incidence of deformed fish [4,5]. Dietary factors, particularly phosphorus concentrations, are also expected to have contributed to the differences in the incidence of skeletal abnormalities observed in the various studies. In this regard, Fjelldal et al. [7] reported that the incidence of deformed vertebrae in triploid salmon was higher than in diploids when the fish were fed diets containing 7.1 (Diploids 35%, Triploids 56%) or 9.4 (Diploids 17%, Triploids 45%) g phosphorus kg^-1^ diet, but not when the diet contained 16.3 g phosphorus kg^-1^ diet (Diploids 8%, Triploids 10%). Even though the diets used in the present study had a higher phosphorus concentration (ca. 18 g kg^-1^) than that reported to mitigate vertebral deformities in triploid salmon [7] the proportions of fish with vertebral anomalies were higher amongst the triploids than diploids. This was the case even though phosphorus bioavailability appeared to be high for fish of both ploidies (Table 3), irrespective of dietary treatment. The percentage of triploid salmon with vertebral anomalies was lower for those fed the HFM diet than those given the STD diet. It is possible, but unlikely, that this can be explained by the differences in phosphorus ADCs between the diets, because phosphorus ADC was lower for the STD diet (Table 3). The proportions of triploid fish with vertebral anomalies in our study were higher than reported by Fjelldal et al. [7], possibly reflecting differences in the genetic backgrounds of the fish, the conditions employed to induce triploidy, or differences in dietary mineral balances and bioavailability between the two studies.

Contrary to expectation the inclusion of fish hydrolysate in the diet did not result in any improvement in the ADC of protein for either diploids or triploids. In addition, the ADC of protein in both diets was lower for triploids than diploids, so the inclusion of fish hydrolysate was not effective in alleviating the differences in protein bioavailability observed in the fish fed the STD diet.

There were no clear and consistent differences in growth between the diploid and triploid salmon in the present study (Fig 4 and Table 2), although the condition factor of the triploids was generally lower than that of diploids throughout the trial (Fig 4 and S2 Table). These findings are largely in agreement with observations made previously [2,11]. Although triploids may be smaller than diploids at the time of hatch and start-feeding, any differences tend to disappear with the passage of time, leading to the diploid and triploid fish being of similar size at the time of the parr-smolt transformation [5,23–26]. On the other hand, Fjelldal et al. [7] reported that phosphorus-rich diets promoted the growth of triploid salmon to a greater extent than diploids, and that triploids fed phosphorus–rich diets were larger at parr-smolt transformation than their diploid counterparts. This trend was also seen in the present study, with the triploid salmon given the STD diet being significantly larger than the corresponding diploids at parr-smolt transformation (Fig 4 and S2 Table). Thus, it appears that triploid salmon must be fed a diet that is more phosphorus-rich than current recommendations for salmon [17] in order to fulfil their growth potential.

The results of the present study indicate that the incidence of skeletal deformities is low when juvenile salmon are fed high-protein phosphorus-rich diets in combination with low egg incubation and rearing temperatures. Such an outcome was predicted on the basis of the results of previous work that demonstrate the separate effects that dietary phosphorus concentrations and rearing temperature can have on the development of skeletal abnormalities in juvenile salmon [4,5,7]. It was interesting to note that feeding a diet with hydrolysed fish protein (HFM) appeared to give a marked reduction in the incidence of vertebral anomalies in triploid salmon compared with triploids fed the phosphorus-rich STD diet that lacked the hydrolysate. To what extent this relates to differences in phosphorus bioavailability between the two diets is a matter of speculation.

Although the use of phosphorus-rich diets may be beneficial for the production and welfare of triploid salmon during the freshwater rearing phase, there are also potential negative consequences of using such diets [27]. The use of phosphorus-rich diets will increase both faecal and non-faecal excretion of phosphorus, leading to increased phosphorus concentrations in the effluent water that leaves the fish rearing units [27]. Phosphorus is a limiting element for primary production in fresh water, and high levels of phosphorus present in the effluent released from fish farms contribute to the eutrophication of recipient freshwater lakes, streams and rivers [27,28]. Consequently, when phosphorus-rich diets are used for the farming of fish in fresh water, some form of wastewater treatment [29] will be required to recover a high proportion of the phosphorus from the fish farm effluent.

## Supporting information

S1 Table Fish deformities. Vertebral numbers, skeletal deformities and abnormalities of diploid (2n) and triploid (3n) Atlantic salmon, *Salmo salar*, fed a fish meal (STD) diet and a diet containing high proportions of hydrolyzed fish protein (HFM) at parr (1390 ddPSF) and smolt (2745 ddPSF) stage. Different superscript letters denote significant differences (P<0.05). Data are presented as means ± SEM (n = 3).
(TIF)

S2 Table Fish growth. Body weight, length and condition factor (K factor) of diploid (2n) and triploid (3n) Atlantic salmon, Salmo salar, fed a fish meal (STD) diet and a diet containing high proportions of hydrolyzed fish protein (HFM) at ten sampling points in the period 95–2745 degree-days post start-feeding (ddPSF). Different superscript letters denote significant differences (P<0.05). Data are presented as means ± SEM (n = 3).
(TIF)
