# Supplementary figures and images for: Growth and development of skeletal anomalies in diploid and triploid Atlantic salmon (Salmo salar) fed phosphorus-rich diets with fish meal and hydrolyzed fish protein
Source: PLoS One. 2018 Mar 22;13(3):e0194340. doi: 10.1371/journal.pone.0194340 (PMC5864013; doi:10.1371/journal.pone.0194340)

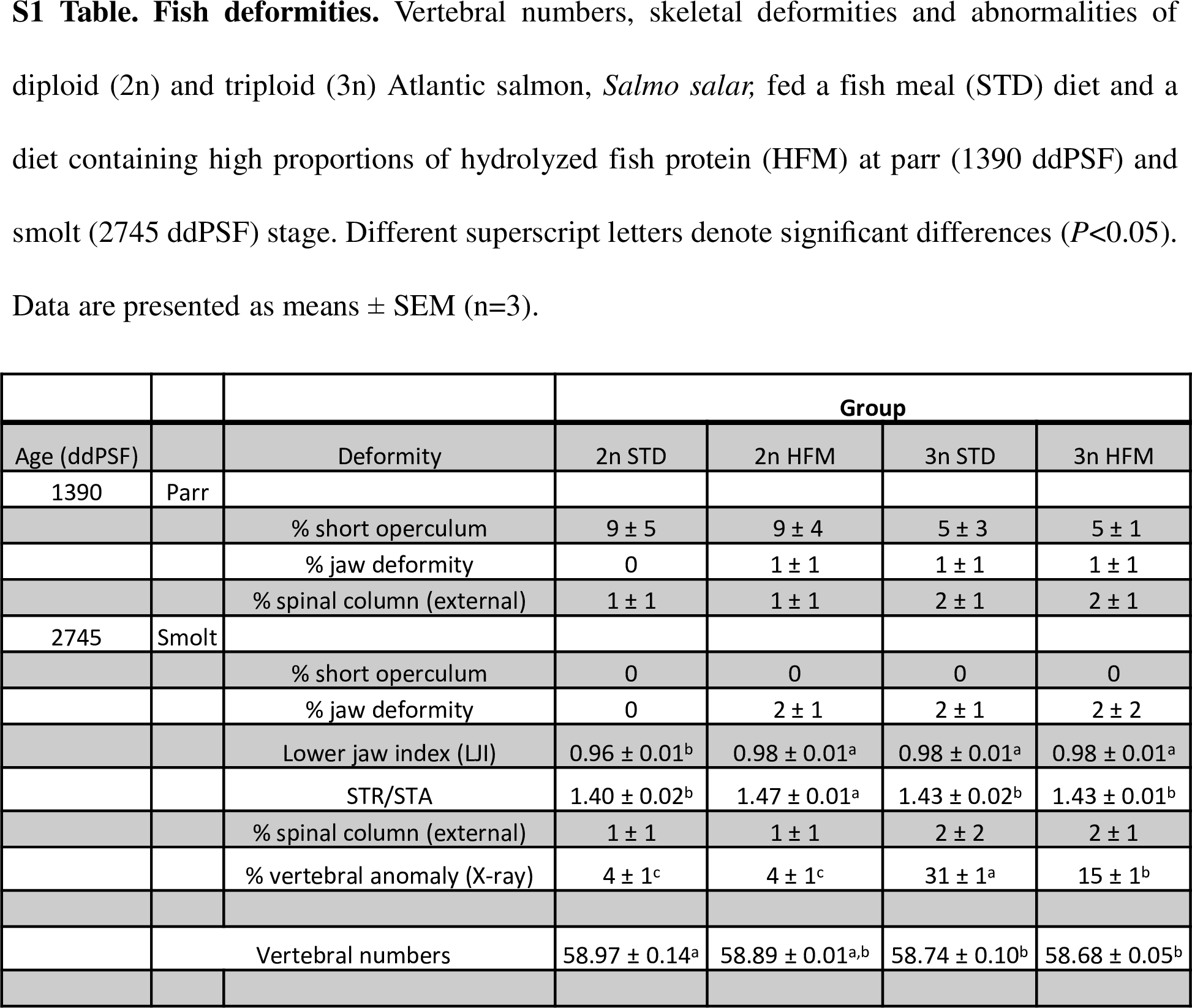

Supplement: S1 Table — Vertebral numbers, skeletal deformities and abnormalities of diploid (2n) and triploid (3n) Atlantic salmon, Salmo salar, fed a fish meal (STD) diet and a diet containing high proportions of hydrolyzed fish protein (HFM) at parr (1390 ddPSF) and smolt (2745 ddPSF) stage. Different superscript letters denote significant differences (P<0.05). Data are presented as means ± SEM (n = 3). (TIF) [file pone.0194340.s001.tif]

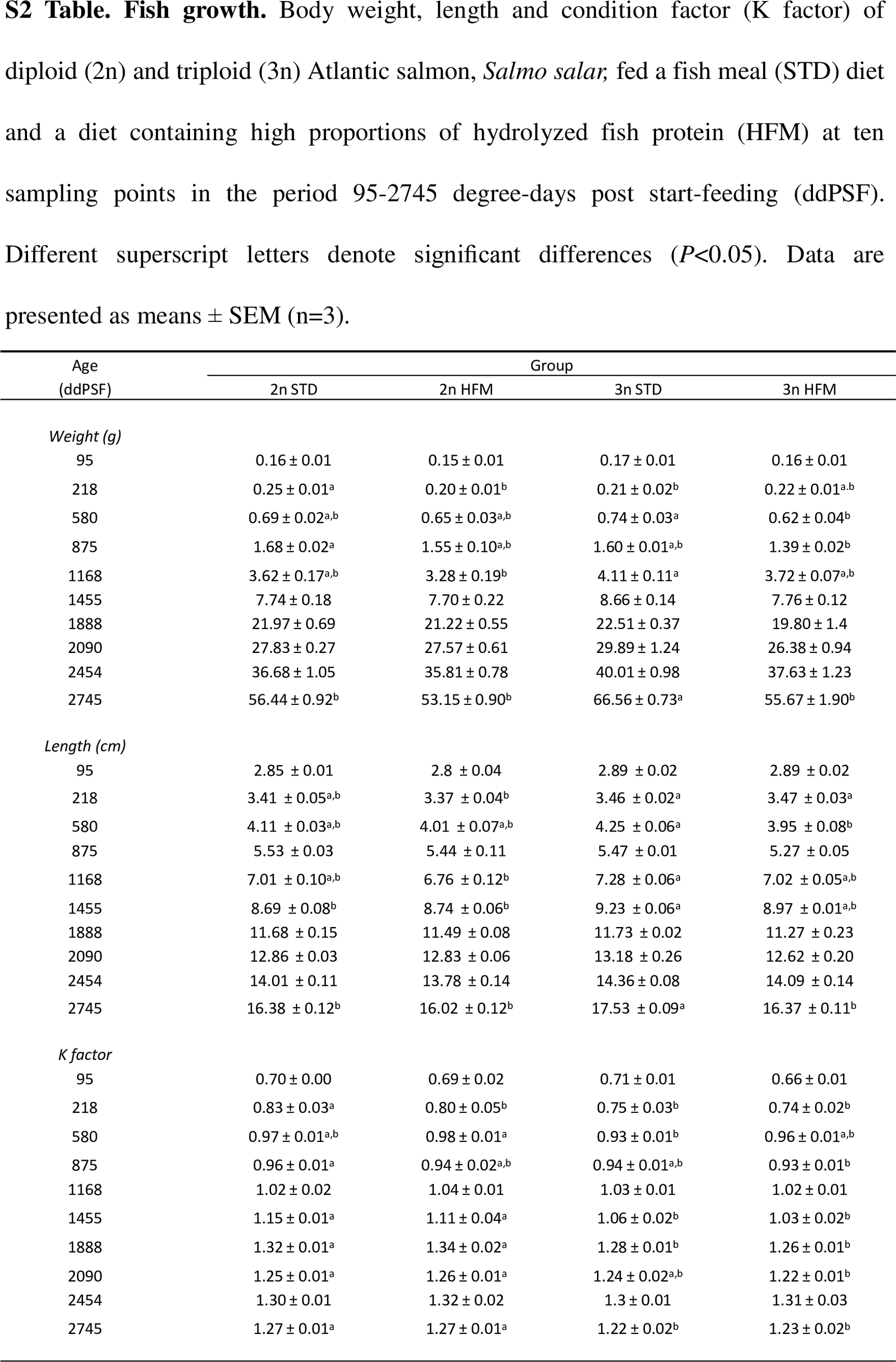

Supplement: S2 Table — Body weight, length and condition factor (K factor) of diploid (2n) and triploid (3n) Atlantic salmon, Salmo salar, fed a fish meal (STD) diet and a diet containing high proportions of hydrolyzed fish protein (HFM) at ten sampling points in the period 95–2745 degree-days post start-feeding (ddPSF). Different superscript letters denote significant differences (P<0.05). Data are presented as means ± SEM (n = 3). (TIF) [file pone.0194340.s002.tif]
